# Authenticity and brain health: a values-based perspective and cultural education approach

**DOI:** 10.3389/fneur.2023.1206142

**Published:** 2023-08-01

**Authors:** Lucy E. Stirland, Biniyam A. Ayele, Catherine Correa-Lopera, Virginia E. Sturm

**Affiliations:** ^1^Global Brain Health Institute, University of California, San Francisco, CA, United States; ^2^Centre for Clinical Brain Sciences, University of Edinburgh, Edinburgh, United Kingdom; ^3^Department of Neurology, College of Health Sciences, Addis Ababa University, Addis Ababa, Ethiopia; ^4^Memory and Aging Center, University of California, San Francisco, CA, United States

**Keywords:** authenticity, resilience, brain health, dementia, values

## Abstract

This perspective paper discusses the concept of authenticity in relation to brain health and neurodegenerative diseases. We define authenticity as being true to oneself and consider it a social value of relevance to neuroscientists, clinicians, and caregivers. From a biological perspective, behaviors that can be interpreted as expressions of authenticity are produced by distributed brain networks. By understanding it as a dynamic process, we argue that harnessing authenticity across the lifespan can be protective by promoting resilience. We discuss the idea of authentic aging, which appreciates the complexity of human life within the world and can enhance positive views of later life. Authenticity is additionally applicable to caring for people with neurodegenerative diseases, both when understanding the behavior of people with dementia and the response of caregivers. Tailoring care to an individual’s personality and strengths may improve their brain health. Finally, we describe an interdisciplinary learning event, themed around masks, designed to engage participants in identifying authenticity in their own work. For scientists, care professionals, and caregivers, reflecting upon authenticity can aid understanding of the person with dementia and therefore improve care.

## 1. Introduction

Authenticity is a value with direct relevance to brain health. It is defined as the consistency between an entity’s internal values and its external expressions or, simply, being true to oneself (1, 2). Brain health is “a life-long, multidimensional, dynamic state consisting of cognitive, emotional, and motor domains underpinned by physiological processes” (3). In neurodegenerative diseases, where brain health is compromised, the whole person is affected. It is therefore relevant to understand a disease’s impact on their humanity through a values-based perspective.

There has been little work to date on the application of authenticity to brain health, so we aim to summarize key concepts in this area and highlight the relevance of authenticity to both neurobiology and psychology. We then outline an illustrative educational event designed to stimulate reflection on the practical application of authenticity in brain health.

## 2. Neuroscience of authenticity

### 2.1. Authenticity as a social value

Authenticity is primarily a social value. Inherent in its conceptualization are how a person interacts with others, and how authentic others consider their behavior to be (4). In a recent review on authenticity, Dammann et al. summarized the concept in four major categories (4C’s): *consistency, conformity, connection, and continuity* (5). The categories are interconnected and relate authenticity to human behavior and interpersonal relationships. When a person is authentic, they naturally exhibit consistency in their behavior. They are true to themselves and consistently act in ways that reflect their genuine beliefs, values, and inner self. Authenticity and conformity can sometimes conflict with each other. In situations where conformity is valued, individuals may feel compelled to suppress their authentic selves to fit in or be accepted by the group. However, maintaining authenticity while navigating social norms is essential for a healthy sense of self. Connection requires genuine communication, empathy, and vulnerability. Authenticity plays a vital role in establishing meaningful interpersonal relationships (6).

Authentic individuals tend to exhibit continuity in their lives by staying true to their core values and beliefs. They navigate changes and challenges while remaining grounded in their authentic selves. In this 4C’s model, continuity indicates that authenticity is inherently a non-static value that allows for changes in authenticity over time and places (5). Therefore, we surmise that authenticity is a dynamic process amenable to development. With self-reflection and by discussing important core values with others, we can better understand ourselves and improve our awareness and practice of our own authenticity. To our knowledge this is an area that has not yet been studied but has potential for research with a view to improving resilience.

### 2.2. Neurobiology of authenticity

To date, most research into authenticity has focused on psychology and individual differences, with little empirical evidence of its localization in the brain, especially in relation to neurodegeneration (7). However, a recent structural magnetic resonance imaging (sMRI) study of 112 healthy young adult volunteers measured trait authenticity using the Authenticity Scale, which contains three sections: self-alienation (awareness of one’s physiological states, emotions, and cognitions), authentic living (congruence between one’s behavior and one’s physiological states, emotions, and cognitions), and accepting external influence (the extent to which one believes they must conform to others’ expectations) (7, 8). The investigators found that higher scores on the Authenticity Scale were associated with a larger left precuneus surface area and that lower self-alienation scores (as an indicator of higher authenticity) were associated with smaller volumes of the left amygdala (7). These are key default mode regions for supporting self-awareness (9). The authors also reported that authenticity mediated the relationship between these brain morphological characteristics and self-reported anxiety, suggesting a protective effect of perceived authenticity (how authentic a person feels) (10) on brain health.

### 2.3. Neurobiology of self-awareness

In the absence of other neurobiological examinations of authenticity, it can be understood through the related concept of self-awareness. As authenticity relies upon the degree of consistency and congruency between the inner self and the projected behavior, it is dependent on self-awareness. Self-awareness is mediated by complex neural circuits that receive both somatic and proprioceptive sensory outputs from the environment (9, 11). It can be divided into two: awareness of the minimal self and of the longitudinal self. The minimal self refers to our physical self in the present moment and includes the current condition of our body, for example our facial features and body position. It also includes the states of our visceral organs and mind and as well as our behavior. Minimal self-awareness is primarily guided by somatic and interoceptive inputs that are represented in brain systems anchored by the somatosensory cortex and insula, respectively. Afferent pathways from the body relay information from the organs and muscles via the laminaI spinothalamocortical tract and vagal afferents to the brainstem, thalamus, and onward to the posterior, mid-, and anterior insula where they are integrated with ongoing contextual details (12). These internal cues color subjective experience and guide decision-making and behavior.

The longitudinal self refers to our continuous existence as a being over time and is primarily built of semantic self-knowledge and autobiographical memories (9). Unlike the minimal self, the longitudinal self is supported by the semantic appraisal network, which has hubs in the anterior temporal lobes and connections to the ventromedial prefrontal cortex, nucleus accumbens, amygdala, and subgenual anterior cingulate cortex, and the default mode network, which includes the ventromedial prefrontal cortex, medial temporal lobes, precuneus, posterior cingulate cortex, and lateral temporoparietal cortex. The semantic appraisal network shows prominent decline in the sematic variant of primary progressive aphasia, a disorder characterized by progressive loss of conceptual knowledge (13, 14). The default mode network is active at rest and during tasks of autobiographic recall and shows selective dysfunction in Alzheimer’s disease (9, 11).

The neurobiology of authenticity involves a complex interplay between different brain systems, with the prefrontal cortex playing a central role in self-reflection, self-regulation, and the integration of cognitive and emotional processes (15–18). The neuro-localization of authenticity has been primarily associated with the prefrontal cortex (PFC) and its connections with other brain regions (16). The PFC, particularly the ventromedial prefrontal cortex, as discussed in relation to the longitudinal self, plays a crucial role in social cognition, decision-making, self-representation, and the integration of emotional and cognitive processes. Studies have shown that the ventromedial PFC is involved in self-reflection and self-awareness, which are fundamental aspects of authenticity (15, 16). The ventromedial PFC is also involved in processing and integrating emotional information, which is essential for authentic emotional expression and empathy for others. Therefore, dysfunction in these neural circuits could manifest as impaired self-awareness, or diminished insight, in neurodegenerative syndromes such Alzheimer’s disease and frontotemporal dementia (FTD) (15–17).

### 2.4. Authenticity and resilience

Perceived authenticity, that is how authentic an individual perceives themselves to be, has been associated with mental resilience and has been found to be helpful in recovery from individual and collective trauma in younger adults. In a survey of undergraduate students, Maffly-Kipp et al. assessed the role of perceived authenticity among individuals affected by Hurricane Harvey, which struck Texas and Louisiana in August 2017 (10). The investigators assessed perceived authenticity using the Authenticity Scale (8). The authors found that participants who scored lower on the Authenticity Scale 4 weeks after Hurricane Harvey reported greater levels of stress 9 weeks after the hurricane than individuals with higher scores of perceived authenticity.

Furthermore, authentic living has a positive association with numerous markers of psychological wellbeing such as life satisfaction and self-esteem and a negative association with indicators of psychological distress such as anxiety and depression (5, 19). Goldman and Kernis asked psychology students to respond to questions that measured their authenticity and found strong correlations between authenticity and both self-esteem and a composite measure of subjective well-being (20). The inverse relationship between authenticity and distress may reveal a possible mechanism for enhancing resilience in early and mid-adulthood. It also highlights the applicability of the value of authenticity to lifelong brain health. There is, however, currently a lack of studies on authenticity and wellbeing or resilience in older adults.

## 3. Application of authenticity to brain health

### 3.1. Authentic aging and brain health

The relatively new concept of brain health encompasses the bidirectional relationship between the health of the brain and the rest of the body, and the close link between brain health and aging in general (3). The principle of “authentic aging” has been proposed as an alternative to the more biomedical notion of “successful aging” or the potentially loaded “healthy aging” (4). Authentic aging acknowledges the complexity of a human life that is lived within an interconnected world. It allows for growth in later life and accepts that throughout life, including in old age, there are opportunities for increased knowledge and expression of the self (21). Although later life is often considered in relation to its proximity to death, this period can also be a time of wisdom and acceptance (22). If authentic living means being true to oneself and living in accordance with our own values and beliefs, (8) authentic aging encompasses these processes alongside maturity and the richness of life experience. Taking a positive approach to brain aging encourages us to consider the possibility of improving or maintaining brain health by harnessing a person’s lifelong strengths when designing person-centered care. Despite a lack of empirical evidence on the overlap between authenticity and brain health, using these conceptual frameworks can aid the understanding of neurogenerative diseases.

### 3.2. Impairment of self-awareness in neurodegenerative disorders

To live authentically and to be true to ourselves, we first need to know ourselves. Changes in the brain due to aging and neurodegenerative disease can manifest in altered behavior related to reduced self-awareness (23). For some people with Alzheimer’s dementia and the people close to them, the loss of autobiographical memories and changes in behavior can feel like losing part of the self (24). If authenticity means being true to oneself and the self is becoming lost, it may seem that people with Alzheimer’s dementia can only be authentic to their past self. In addition, in people with dementia who experience depression, low self-esteem is a common symptom (25). Self-esteem is closely linked to self-awareness, with some evidence that increased awareness of the self in people with existing low self-esteem can in fact negatively affect mood (26).

In other types of dementia, such as FTD, reduced inhibition and changes in emotions, behavior, and language can lead to the person expressing themselves in a way they had not done before. Personality change is a central feature of the behavioral variant of FTD, and there is evidence that people with behavioral variant FTD lack insight into their current personality (27). This may reflect a difficulty in updating information about themselves (9). Applying the concept of authenticity can be challenging here, because if the person’s new behavior is understood as an expression of their authentic self (i.e., their inner voice without a filter), it may seem inauthentic because it may not reflect how that person has always behaved and seem unfamiliar to people who know them. This conflict has previously been considered in relation to schizophrenia: if an affected individual’s personality while ill is seen as not an authentic expression of their self, this discounts the importance of the illness on their life and equally, accepting only their previous personality as authentic discounts their present lived experience (28).

Impaired self-awareness can also be observed in patients with moderate to severe Alzheimer’s dementia. This typically takes a form of overestimation of their cognitive performance, functional abilities, and social behavior. In practice, therefore, it is important to integrate reports from people with dementia with a collateral history from a caregiver (16).

### 3.3. Authenticity and stigma

In some instances, authenticity can lead to stigma, for example when individuals are open about their experience of certain health conditions. Conversely, when a high-profile person feels able to disclose a diagnosis in themselves or their family, their authenticity in doing so can raise awareness of the condition. There is no firm evidence that celebrities talking about personal experiences of dementia reduces stigma and increases diagnosis and treatment rates. However, if a well-known figure publicly accepts and embraces a diagnosis, this may improve perception of the condition (29). Their disclosure can open an opportunity for public education, for example that dementia is not a normal part of aging and that its associated behaviors are due to a physical disease and not personal choice.

### 3.4. Authenticity in dementia care

Building on the biological framework, the behavior of a person with dementia can be more broadly appraised in the context of their personal history and strengths, and their relationships with other people. In all types of dementia, the concept of authenticity can be employed to help understand the person and to improve care. When there is a perceived loss of contact with the self, recognizing and upholding the person’s ability to be authentic can maintain aspects of their personhood (30). For example, especially in residential care settings, where a person may lose touch with tangible aspects of their personal life, supporting their interests can nurture them (31). Regarding the specific symptom of low self-esteem, visual arts education has been found to be an effective intervention (32). In a qualitative study of art, authenticity, and citizenship, the authors argued that the self persists despite cognitive impairment or dementia and that a participatory artistic intervention may promote authentic living in people with dementia in care homes (33).

Authenticity is relevant in assessing decision-making ability in people with dementia. Although legal frameworks for capacity differ by country, most include guidance about honoring the previous views of a person who has lost capacity. In this context, authenticity can apply to a decision being made that is in keeping with their values, previous decisions, life history, and personality (34, 35). It can mean that even when the person is unable to make their own choices, an authentic decision can still be made with them, or on their behalf.

It may also be helpful for professional and informal caregivers to apply or reflect upon the value of authenticity. The Person-Centered Practice Framework outlines that person-centered healthcare should uphold the patient’s own values and also be grounded in professionals’ self-awareness (36). Conversely, it has been argued that in certain situations, the caregiver may have to prioritize the authenticity of the person with dementia over their own (37). For example, the person’s authentic experience on a given day may be disconnected from the caregiver’s reality; they may be waiting or searching for a family member whom the caregiver knows to be deceased. It is common for caregivers in this situation to avoid telling the truth to prevent additional distress (38). Having the ability to discuss challenging ethical questions in relation to authenticity, both before and after they arise, could improve the confidence of professionals and caregivers in coping with these situations.

## 4. Teaching the value of authenticity: a cultural education approach

We defined authenticity as “being true to ourselves.” To explore this in the context of practical application in brain health, we developed a workshop entitled “Behind the Mask.” The participants were faculty and fellows in the Atlantic Fellowship for Equity in Brain Health program at the Global Brain Health Institute (GBHI) at the University of California, San Francisco. The workshop was divided into four sections: an introduction to the concept of authenticity, the neuroscience behind it, its cultural background, and an artistic activity to allow creative group reflection. We offer a brief description of this event so that it might serve as a model for teaching about authenticity.

**Figure 1:**
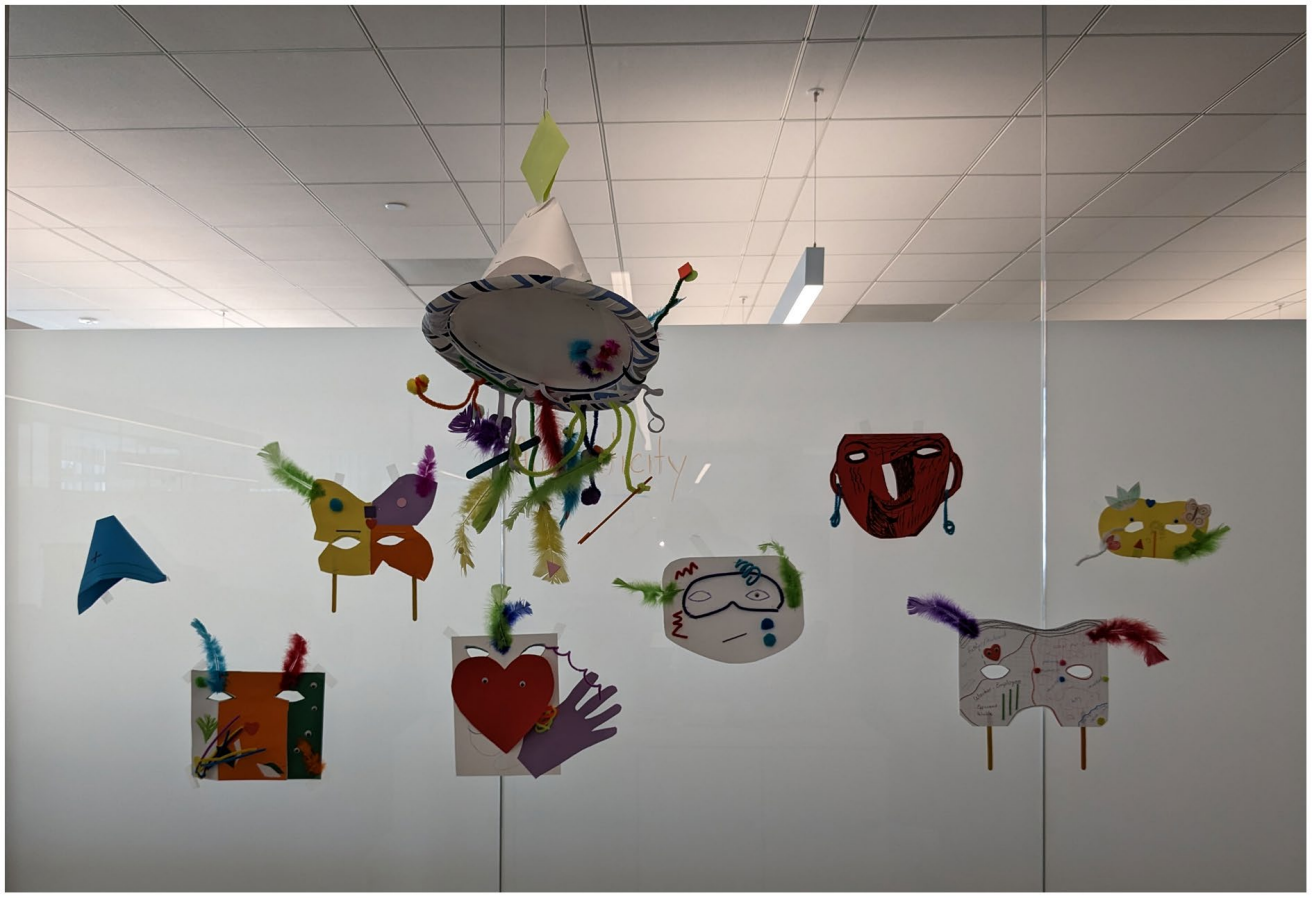
Image of the symbolic masks produced in the workshop.

We aimed to encourage discussion about authenticity in dementia, both relating to the experience of the person with dementia and those who interact with them. To stimulate reflection, we posed the question whether it is possible to be authentic while wearing a mask. Throughout the history of human society, masks have been used for celebration, religion, identity, and as a mark of cultural heritage (39). They allow a person to change their outward expression while they remain the same inside. We asked participants to reflect upon the roles played by two fictional characters: a spouse of a person with dementia and a healthcare worker. Attendees were invited to create masks together to express their thoughts about the various masks that these individuals, and each of us, might wear as we navigate our daily lives. Art helped participants move from the abstract of thinking and talking to the concrete nature of a mask as an object full of colors and shapes. Figure 1 is an image of the symbolic masks produced in the workshop.

Our educational event aimed to encourage the diverse attendees to reflect on authenticity as it related to themselves and to the person affected by dementia. Self-awareness and group reflection can enhance resilience and maintain compassion (40). If caregivers of older people apply concepts of authenticity to both understanding the person for whom they care and to their own experience in a caring role, this may improve resilience and prevent burnout.

## 5. Conclusion

The concept of authenticity encompasses the neurobiological process of self-awareness as well as its psychological manifestations in interpersonal interactions. Adopting a broad translational view of authenticity can offer opportunities for more research on the neurobiological pathways involved, and on potential psychological interventions. We gained value from an educational workshop reflecting on this topic, which would also be of relevance to healthcare practitioners and caregivers of people with dementia.

## Data availability statement

The original contributions presented in the study are included in the article/supplementary material, further inquiries can be directed to the corresponding author.

## Author contributions

LS, BA, and CC-L: conceptualization, writing–original draft, and writing–review and editing. VS: writing–review and editing and supervision. All authors contributed to the article and approved the submitted version.

## Funding

LS, BA, and CC-L are Atlantic Fellows for Equity in Brain Health at the Global Brain Health Institute and did not receive additional funding for this work. VS is supported by National Institutes of Health award number R01AG073244.

## Conflict of interest

The authors declare that the research was conducted in the absence of any commercial or financial relationships that could be construed as a potential conflict of interest.

## Publisher’s note

All claims expressed in this article are solely those of the authors and do not necessarily represent those of their affiliated organizations, or those of the publisher, the editors and the reviewers. Any product that may be evaluated in this article, or claim that may be made by its manufacturer, is not guaranteed or endorsed by the publisher.
